# Ionic Liquid-Based Ultrasonic-Assisted Extraction of Secoisolariciresinol Diglucoside from Flaxseed (*Linum usitatissimum* L.) with Further Purification by an Aqueous Two-Phase System

**DOI:** 10.3390/molecules201017929

**Published:** 2015-09-30

**Authors:** Zhi-Jian Tan, Chao-Yun Wang, Zi-Zhen Yang, Yong-Jian Yi, Hong-Ying Wang, Wan-Lai Zhou, Fen-Fang Li

**Affiliations:** 1 Institute of Bast Fiber Crops, Chinese Academy of Agricultural Sciences, Changsha 410205, China; E-Mails: ibfcyyj@163.com (Y.-J.Y.); Cswhy328@126.com (H.-Y.W.); aruofly@126.com (W.-L.Z.); 2 College of Chemistry and Chemical Engineering, Central South University, Changsha 410083, China; E-Mails: yzz8582352@163.com (Z.-Z.Y.); lfflqq@csu.edu.cn (F.-F.L.)

**Keywords:** ionic liquid, ultrasonic-assisted extraction, aqueous two-phase system, secoisolariciresinol diglucoside, purification

## Abstract

In this work, a two-step extraction methodology of ionic liquid-based ultrasonic-assisted extraction (IL-UAE) and ionic liquid-based aqueous two-phase system (IL-ATPS) was developed for the extraction and purification of secoisolariciresinol diglucoside (SDG) from flaxseed. In the IL-UAE step, several kinds of ILs were investigated as the extractants, to identify the IL that affords the optimum extraction yield. The extraction conditions such as IL concentration, ultrasonic irradiation time, and liquid–solid ratio were optimized using response surface methodology (RSM). In the IL-ATPS step, ATPS formed by adding kosmotropic salts to the IL extract was used for further separation and purification of SDG. The most influential parameters (type and concentration of salt, temperature, and pH) were investigated to obtain the optimum extraction efficiency. The maximum extraction efficiency was 93.35% under the optimal conditions of 45.86% (*w*/*w*) IL and 8.27% (*w*/*w*) Na_2_SO_4_ at 22 °C and pH 11.0. Thus, the combination of IL-UAE and IL-ATPS makes up a simple and effective methodology for the extraction and purification of SDG. This process is also expected to be highly useful for the extraction and purification of bioactive compounds from other important medicinal plants.

## 1. Introduction

Flaxseed is the richest food source of lignans, a plant secondary metabolite, especially secoisolariciresinol diglucoside (SDG), compared to those found in other plants [1,2,3,4]. It also contains omega-3 (ω-3/*n*-3) polyunsaturated fatty acids (specifically α-linolenic acid, ALA), short chain polyunsaturated fatty acids (PUFA) [5,6], a high level of dietary fibers, and good quality protein fractions [7]. In recent years, SDG has attracted increasing interest owing to its pharmacological action, including antidiabetic effects [8], antioxidant activities [9], inhibition of cancer cell growth [10], inhibition of human breast tumor growth [11], and its hypocholesterolemic and antiatherosclerotic effects [12].

Ionic liquids (ILs) are molten salts composed entirely of ions that are in the liquid state at or below 100 °C [13]. Compared with water and conventional organic solvents, they exhibit distinct physical, chemical, and biological properties such as negligible volatility, a wide electrochemical window, high thermal and chemical stability, exceptional solubility, and antimicrobial and analgesic properties [14,15,16]. As green solvents, ILs have been widely used in the extraction of active ingredients from natural plants, such as isoflavones from radix puerariae [17], luteolin from peanut shells [18], saponins and polyphenols from mate (*Ilex paraguariensis*) and tea (*Camellia sinensis*) [19], saponins from sisal (*Agave sisalana*) and juá (*Ziziphus joazeiro*) [20], and flavonoids from *Chamaecyparis obtusa* leaves [21]. Ultrasound-assisted extraction (UAE) is a common auxiliary method used in the extraction field. UAE can break down the plant tissue more easily and accelerate the penetrating of solvent into the plant tissue, reduce processing time and energy and enhance efficiency in mass and energy transfer [22,23]. Ionic liquid-based ultrasonic-assisted extraction (IL-UAE) has been extensively demonstrated in the efficient extraction of active ingredients from natural plants, such as forskolin from *Coleus forskohlii* roots [24], isoliquiritigenin, liquiritin and glycyrrhizic acid from licorice [25], and alkaloids from *Phellodendron amurense* Rupr [26]. 

An ionic liquid aqueous two-phase system (IL-ATPS) was reported for the first time in 2003 by Rogers and coworkers [27]. The advantages of this system include negligible viscosity, formation of very little emulsion, no need of using volatile organic solvent, and gentle biocompatible conditions of use [28,29,30]. Up to now, IL-ATPS has been successfully used in the extraction and purification of different compounds such as polysaccharides and proteins [31], gallic acid [32], sulfonamides [28], cephalexin [33], and wheat-esterase [34].

In this paper, we report a two-step extraction using IL-UAE and IL-ATPS to extract and purify SDG from flaxseeds. Five kinds of ILs were considered as the IL-UAE extractant. SDG was further enriched and purified using IL-ATPS. The extraction conditions of the IL-UAE (*i.e.*, IL concentration, ultrasonic irradiation time, and liquid–solid ratio) were optimized using the response surface methodology (RSM). The most influential parameters in IL-ATPS (type and concentration of salt, temperature, and pH) were also investigated.

## 2. Results and Discussion

### 2.1. Ionic Liquid-Based Ultrasonic-Assisted Extraction (IL-UAE)

#### 2.1.1. Selection of IL

**Figure 1:**
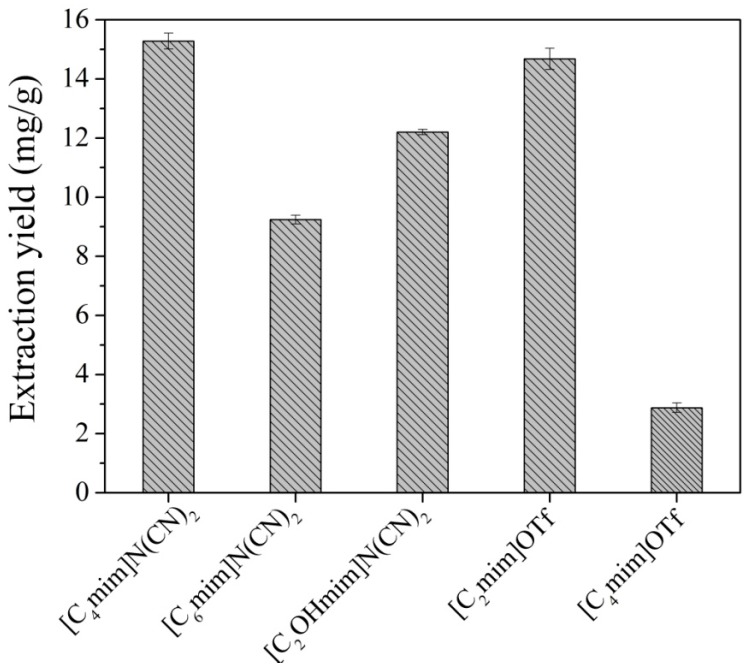
Ionic liquids-based ultrasound-assisted extraction of SDG using five types of ILs.

In order to find the optimal extractant, five types of ILs were investigated for the extraction of SDG from flaxseed powder. Although the pure ILs selected for extraction have low viscosity, some studies reported that despite that ILs normally possesses high viscosity, the presence of water reduces it considerably [35,36]. Therefore, the viscosity of pure ILs is less important when the ILs aqueous solutions are used in the solid-liquid extraction. The results in Figure 1 show that the IL [C_4_mim]N(CN)_2_ led to the optimum extraction yield. The extraction ability might be related to the hydrophobicity of ILs, as the extraction ability of ILs increases with the increase of IL hydrophobicity. The hydrophobicity of the ILs used in this study follow the order: [C_2_mim]OTf < [C_2_OHmim]N(CN)_2_ < [C_4_mim]N(CN)_2_ < [C_6_mim]N(CN)_2_ < [C_4_mim]OTf. However, as to [C_4_mim]N(CN)_2_ and [C_4_mim]OTf, because of different solutions have different multi-interactions including π-π, n-π, ionic/charge-charge and hydrogen bonding with SDG of interest, which results in the decrease of extraction yield for [C_4_mim]OTf [37,38]; as to [C_4_mim]N(CN)_2_ and [C_6_mim]N(CN)_2_, increasing the alkyl chain length from butyl to hexyl decreases the extraction efficiency, which could be attributed to increased steric hindrance with the longer alkyl chain [39]. Therefore, this IL [C_4_mim]N(CN)_2_ has the optimal extraction ability and was chosen as the extractant for further study.

#### 2.1.2. Single Factor Experiments

A number of factors, including IL concentration, liquid–solid ratio, and ultrasonic irradiation time, could affect the UAE extraction yield. Therefore, it is necessary to identify the most influential factors for obtaining the maximum extraction yield. 

##### Effect of IL Concentration

**Figure 2:**
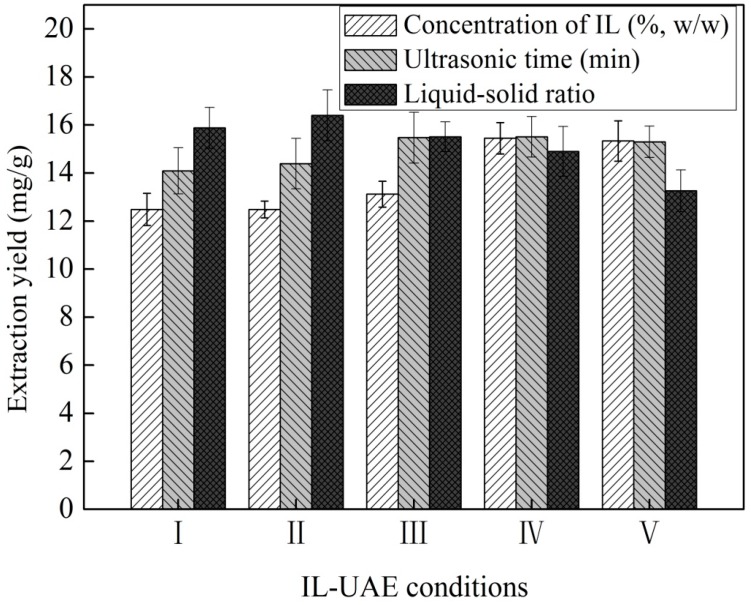
Single-factor experiments of IL concentration (I–V represent the IL concentrations of 20%, 30%, 40%, 50%, and 60% (*w*/*w*)),ultrasonic irradiation time (I–V represent the ultrasonic irradiation time of 20, 30, 40, 50, and 60 min), and liquid–solid ratio (I–V represent the liquid–solid ratio of 10:1, 20:1, 30:1, 40:1, and 50:1) for the extraction of SDG using IL-UAE.

The effect of [C_4_mim]N(CN)_2_ aqueous solutions with mass concentrations of 20%–60% (*w*/*w*) was calculated to determine the optimal concentration of IL-UAE. The liquid–solid ratio and ultrasonic irradiation time were set at constant values of 20:1 and 30 min, respectively. The maximum extraction yield was obtained at IL concentration of 50% (*w*/*w*) (Figure 2). No obvious increase in the extraction yield was observed with further increase in the IL concentration; thus, the IL concentration of 50% (*w*/*w*) was selected for subsequent experiments.

##### Effect of Liquid–Solid Ratio

The liquid–solid ratio is an important parameter influencing the extraction yield. If the liquid–solid ratio is too high, the result would be waste of IL and increased cost, while a too-low ratio would result in incomplete extraction. The effect of liquid–solid ratios of 10:1, 20:1, 30:1, 40:1, and 50:1 was studied to evaluate the influence of this ratio on extraction yield. The IL concentration and ultrasonic irradiation time were set at constant values of 50% (*w*/*w*) and 30 min, respectively. The results in Figure 2 showed that the maximal extraction yield was obtained when the liquid–solid ratio was 20:1.

##### Effect of Ultrasonic Irradiation Time

To some extent, the ultrasonic irradiation time also plays an important role in the IL-UAE procedure, because sonication significantly shortens the extraction time. The effect of ultrasonic irradiation time from 20 to 60 min was studied by maintaining other experimental conditions constant such as IL concentration of 50% (*w*/*w*) and liquid–solid ratio of 20:1. Higher extraction yields were obtained when the ultrasonic irradiation time was longer than 40 min; no obvious increase was observed with further increase in time (Figure 2).

#### 2.1.3. Optimization of IL-UAE Conditions by the Response Surface Method (RSM)

To further study the interaction between these factors, the conditions were optimized using RSM in Design-Expert 7.0 software (Stat-Ease, Minneapolis, MN, USA). Three factors: IL concentration (40%, 50%, and 60% (*w*/*w*)), liquid–solid ratio (10:1, 20:1, and 30:1), and ultrasonic irradiation time (40, 50, and 60 min), were applied using a Box–Behnken design. The experimental conditions are shown in Table 1. Experimental results of the extraction yield of SDG were analyzed by multiple regressions to fit the second-order regression equation. The coded factors of the regression model were predicted to be: Y = 15.8 + 0.66A + 0.63B + 0.12C + 0.85AB − 0.21AC − 0.24BC − 1.15A^2^ − 1.48B^2^ + 0.49C^2^ (R^2^ = 0.9883), where Y is the extraction yield in mg/g, A is the IL concentration (%, *w*/*w*), B is the liquid–solid ratio, and C is ultrasonic irradiation time (min).

**Table 1:** Arrangement and results of the three-factor/three-level response surface design.

Run	Factor A: IL Concentration (%, *w*/*w*)	Factor B: Liquid–Solid Ratio	Factor C:Ultrasonic Irradiation Time (min)	Response Average Extraction Yield (mg/g)
1	60	10:1	50	12.5521
2	50	20:1	50	15.7939
3	50	20:1	50	15.9827
4	60	30:1	50	15.4370
5	50	10:1	40	13.7683
6	40	30:1	50	12.0817
7	50	30:1	60	15.3657
8	50	10:1	60	14.5096
9	50	30:1	40	15.5716
10	40	10:1	50	12.6094
11	60	20:1	60	15.5444
12	60	20:1	40	15.7341
13	50	20:1	50	15.8536
14	50	20:1	50	15.7837
15	40	20:1	40	14.3129
16	50	20:1	50	15.5716
17	40	20:1	60	14.9624

It can be seen in Table 2 that the value for the coefficient of determination (R^2^) was 0.9883, which implies that over 98.83% of the variation in the process efficiency could be explained by the model. The model F-value of 65.53 and the model *p*-value of <0.0001 indicate that the model is significant. There is only less than 0.01% chance that such a large “Model F-Value” could occur due to noise, implying that the model was acceptable. A “Prob > F” of value less than 0.0500 indicates that the model terms are significant. In this case, A, B, AB, A^2^, B^2^, and C^2^ are significant model terms.

**Table 2:** Analysis of variance (ANOVA) for the quadratic response surface model.

Source	Degrees of Freedom	Sum of Squares	Mean Square	F-value	*p*-value Prob > F
Model	9	26.15	2.91	65.53	<0.0001
A	1	3.51	3.51	79.23	<0.0001
B	1	3.15	3.15	70.95	<0.0001
C	1	0.12	0.12	2.79	0.1386
AB	1	2.91	2.91	65.66	<0.0001
AC	1	0.18	0.18	3.97	0.0865
BC	1	0.22	0.22	5.06	0.0595
A2	1	5.53	5.53	124.76	<0.0001
B2	1	9.23	9.23	208.24	<0.0001
C2	1	1.00	1.00	22.57	0.0021
Residual	7	0.31	0.044		
Lack of fit	3	0.22	0.074	3.33	0.1376
Pure Error	4	0.089	0.022		
Cor total	16	26.46			

**Figure 3:**
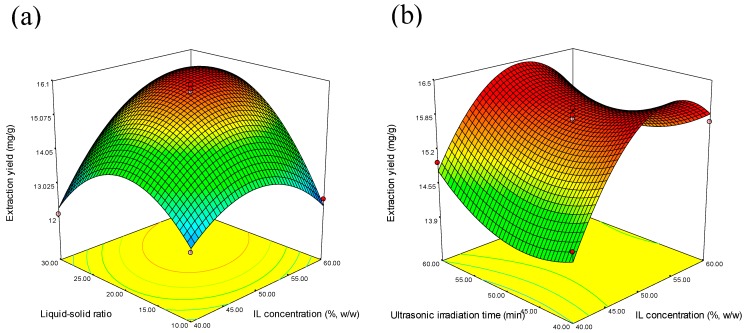

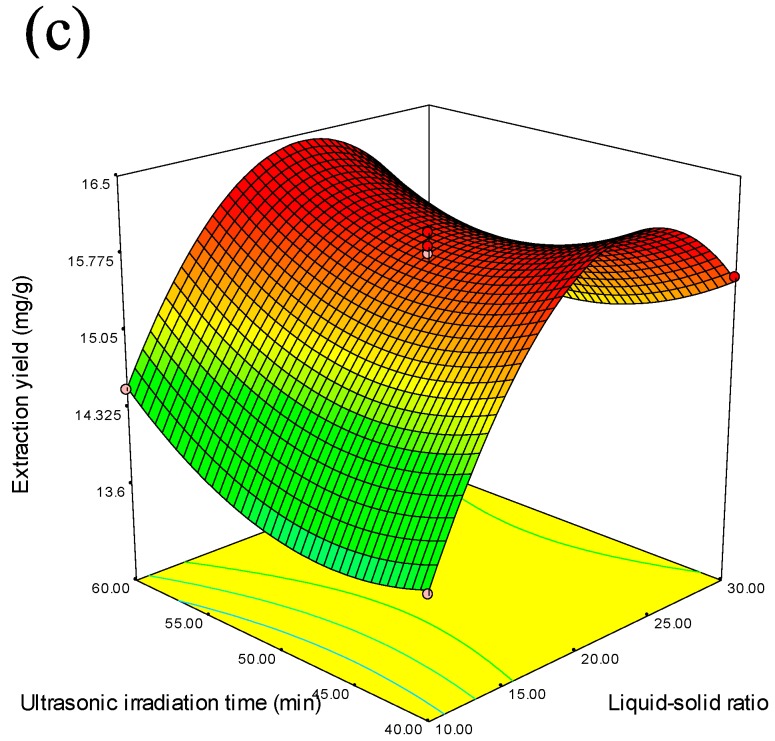
Response surface plots showing the effects of variables on the average extraction yield of SDG: (**a**) interaction of the IL concentration and liquid–solid ratio; (**b**) interaction of the IL concentration and ultrasonic irradiation time; and (**c**) interaction of the liquid–solid ratio and ultrasonic irradiation time.

The response surfaces for the effects of independent variables on the average extraction yield of SDG are shown in Figure 3. Based on the quadratic model, the optimum conditions for the extraction of SDG were calculated to be IL concentration of 55.49% (*w*/*w*), liquid–solid ratio of 24.50:1, and ultrasonic irradiation time of 40 min. Triplicate runs were carried out at the optimum conditions, and the average yield was 16.3374 mg/g, which is very close to the predicted value of 16.5934 mg/g. This demonstrated that the model was adequate for reflecting the expected optimization.

#### 2.1.4. Comparison with Other Extraction Methods

The heat reflux extraction (HRE) and microwave-assisted extraction (MAE) were compared with UAE to extract SDG using [C_4_mim]N(CN)_2_ as extractant. The operating temperature of the three methods was set at approximate 40 °C. The results are presented in Table 3, which shows that the IL-UAE and IL-MAE have higher extraction yield and consume a shorter extraction time than IL-HRE. The IL-MAE give similar yield with IL-UAE, however, MAE gives microwave leakage that is potentially harmful to humans compared with UAE. 

**Table 3:** Comparison of three different methods (HRE, MAE, and UAE) for extraction of SDG (IL concentration is 50% (*w*/*w*) and liquid–solid ratio is 20:1).

Extraction Methods	HRE			MAE	UAE
Time (min)	20	40	60	40	40
Extraction yield (mg/g)	2.6953	9.6581	12.6324	16.1263	16.0854

### 2.2. Ionic Liquid-Based Aqueous Two-Phase System (IL-ATPS)

In the IL-ATPS section, a kosmotropic salt was directly added into the IL extract to construct ATPS for further purification of SDG. The influential parameters: type and concentration of salt, temperature, and pH, were investigated.

#### 2.2.1. Effect of Salt

**Figure 4:**
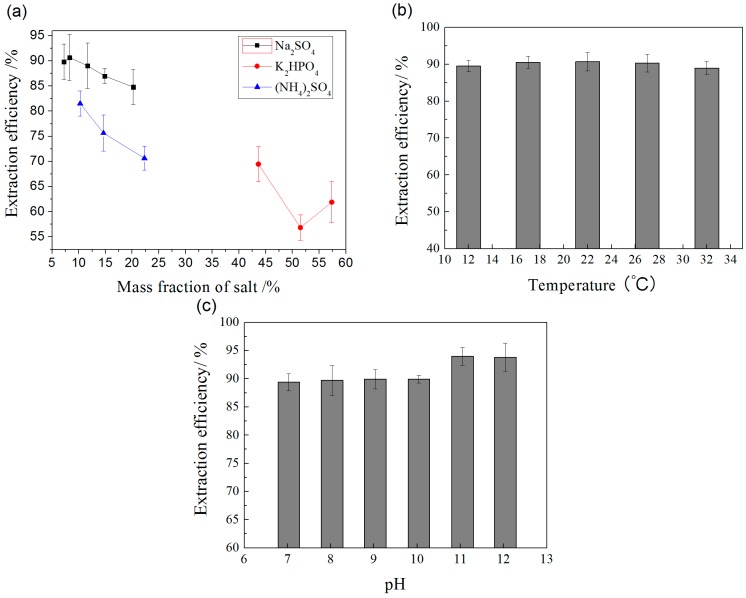
Effect of (**a**) salt; (**b**) temperature; and (**c**) pH on the extraction efficiency of SDG by IL-ATPS.

Three types of salt, an acid salt [(NH_4_)_2_SO_4_], a basic salt [K_2_HPO_4_], and a neutral salt [Na_2_SO_4_], were considered as candidates for the phase-forming salt. It can be seen in Figure 4a that the ATPSs formed by Na_2_SO_4_ had the highest extraction efficiency. The extraction ability of IL-ATPSs follows the phase-forming order of three types of salt: Na_2_SO_4_ > (NH_4_)_2_SO_4_ > K_2_HPO_4_. The maximum extraction efficiency was obtained when the ATPS was composed of 45.86% (*w*/*w*) IL and 8.27% (*w*/*w*) Na_2_SO_4_.

#### 2.2.2. Effect of Temperature

The partitioning of SDG in IL-ATPS within the temperature range 12–32 °C was investigated, with unadjusted pH. The results are shown in Figure 4b. When the temperature was 22 °C, the extraction efficiency was maximum. The higher temperature will be not suitable for the stability for SDG. Moreover, a higher temperature will cause consumption of more energy; therefore, it is better to operate the IL-ATPS closing to the room temperature.

#### 2.2.3. Effect of pH

A buffer solution (Na_2_HPO_4_–H_3_PO_4_) was used to adjust the pH of the ATPS. Na_2_SO_4_ was added into IL-UAE extract to form ATPS. The effect of pH within the range 7.0–12.0 on the extraction efficiency was investigated. After IL-ATPS, SDG was quantified by HPLC after alkaline hydrolysis of the samples in IL-rich phase. As shown in Figure 4c, it can be observed that the highest extraction efficiency was obtained at pH 11.0.

**Figure 5:**
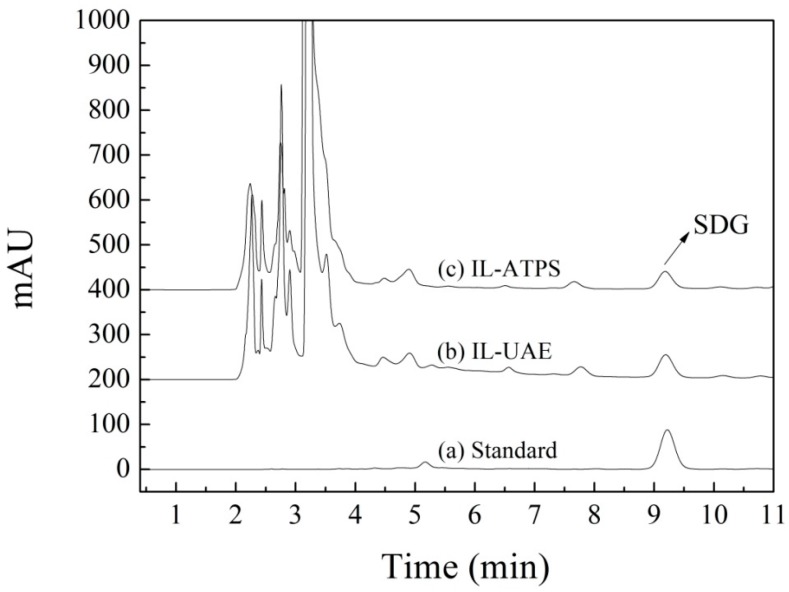
HPLC chromatograms for (a) SDG standard; (b) IL-UAE sample; and (c) IL-ATPS sample.

To conclude, the maximum IL-ATPS extraction efficiency of 93.35% was obtained under the following conditions: ATPS 45.86% (*w*/*w*) [C_4_mim]N(CN)_2_ and 8.27% (*w*/*w*) Na_2_SO_4_, extraction temperature 22 °C, and pH 11.0. Under the optimal IL-UAE and IL-ATPS conditions, the HPLC chromatograms for the analysis of SDG samples after IL-UAE and IL-ATPS are shown in Figure 5.

#### 2.2.4. SDG Isolation and IL Recycling

**Figure 6:**
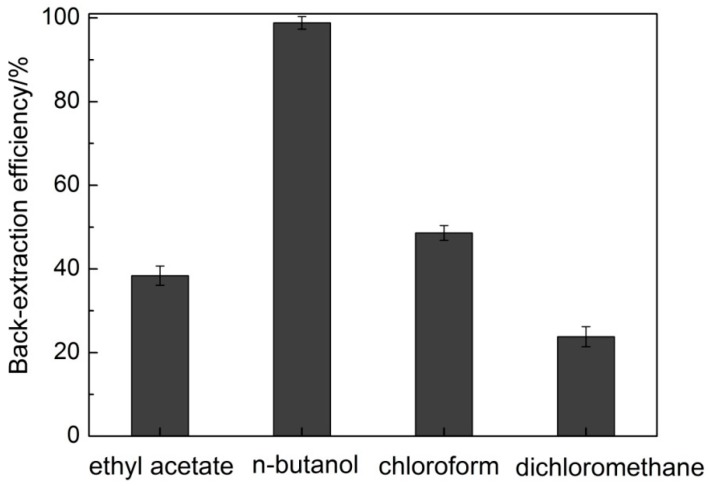
Back-extraction of SDG using organic solvents.

After IL-ATPS, SDG is extracted into IL-rich phase, and how to recovery SDG and IL from the IL-based raw extract is becoming a difficult task. Some literature reported that liquid–liquid back-extraction with organic solvents is a reasonable approach for back-extraction of compounds and recovery of ILs [31,40]. In order to achieve this aim, several non-miscible with water organic solvents of ethyl acetate, *n*-butanol, chloroform, and dichloromethane were tested, to extract SDG into organic phase with IL staying in aqueous phase. SDG can be isolated and IL can be recycled with further treatment after separation of two phases. The results in Figure 6 shows that the maximal back-extraction efficiency of SDG reaches to 98.84% using *n*-butanol as solvent.

## 3. Experimental Section 

### 3.1. Materials and Reagents

Flaxseeds were harvested and collected in August 2014 from Harbin, Heilongjiang Province, Northeast China. The ILs of [C_4_mim]N(CN)_2_ (1-butyl-3-methylimidazoliumte dicyanamide), [C_6_mim]N(CN)_2_ (1-hexyl-3-methylimidazoliumte dicyanamide), [C_2_OHmim]N(CN)_2_, (1-(2-hydroxyethyl)-3-methylimidazolium dicyanamide), [C_2_mim]OTf (1-ethyl-3-methylimidazoliumte triflate), and [C_4_mim]OTf (1-butyl-3-methylimidazoliumte triflate) were synthesized and purified in our laboratory. The viscosity was determined using a Brookfield DV-S viscometer (Middleboro, MA, USA), indicating that the viscosity of all the ILs used in this study was below 50 mPa·s. The standard of SDG (purity >98% by HPLC) was purchased from Shanghai Haling Biological Technology Co., Ltd. (Shanghai, China). HPLC-grade methanol was acquired from TEDIA Company (Fairfield, OH, USA). K_2_HPO_4_, Na_2_SO_4_, and (NH_4_)_2_SO_4_ were of analytical grade and were purchased from Sinopharm Chemical Reagent Co., Ltd. (Shanghai, China). Other reagents were all analytical grade and used without treatment. Deionized water was used to prepare the sample solutions.

### 3.2. Ionic Liquid-Based Ultrasound-Assisted Extraction

The flaxseeds were smashed to powder of about 60 meshes (250 μm) particle size. Then, *n*-hexane was added to the powder (5:1, *v*/*m*) and stirred for about 6 h for degreasing. After evaporating out the *n*-hexane and drying, degreased flaxseed powder was obtained. The IL aqueous solution was added to the degreased flaxseed powder for ultrasonic extraction of SDG in an ultrasonic bath (KQ-5200DE, Kunshan Ultrasound Co. Ltd., Kunshan, China).The electric power is 200 W, generators frequency is 40 kHz, and operating temperature of 25 °C were set for this bath. The IL extract was centrifuged, and then hydrolyzed using NaOH aqueous solutions (0.25–0.4 mol/L) for 2 h. The pH value of the extract solution was adjusted to 4.0–6.0 by HCl aqueous solution before analysis. Actually, SDG is a product of alkaline hydrolysis of polymeric lignans, the IL-UAE extract polymeric compounds without defined structure, which are subsequently hydrolyzed in presence of NaOH. The latter gives a mixture of phenolic compounds, containing a certain amount of SDG. The SDG concentration was quantified using HPLC. The extraction yield of SDG by IL-UAE was calculated according to Equation (1):

(1) $$\text{Extraction yield }\left(\text{mg}/\text{g}\right)\;=\;\frac{Mass\;of\;SDG\;determined\;\left(mg\right)}{Mass\;of\;degreased\;flaxseed\;powder\;\left(g\right)}$$

### 3.3. Ionic Liquids-Based Aqueous Two-Phase System

SDG was extracted from the IL aqueous solution, and a certain amount of the IL-extract and a certain amount of salt were added to a tube. The tube was shaken well to completely dissolve the salt, and then it was centrifuged for complete phase formation. Two clear phases formed, and the volume of each phase was noted. SDG sample in IL-rich phase was withdrawn using a syringe, and then hydrolyzed using NaOH aqueous solutions (0.25–0.4 mol/L) for 2 h. The pH value of the sample was adjusted to 4.0–6.0 by HCl aqueous solution before HPLC analysis. The SDG concentration in the salt-rich phase was determined by mass balance.

The phase ratio (R) was defined as in Equation (2):

(2) $$\text{R}=\frac{{\text{V}}_{\text{t}}}{{\text{V}}_{\text{b}}}$$

where V_t_ and V_b_ are the volumes of IL-rich phase and salt-rich phase, respectively. The partition coefficient (K) was defined as in Equation (3):

(3) $$\text{K}=\frac{{\text{C}}_{\text{t}}}{{\text{C}}_{\text{b}}}$$

where C_t_ and C_b_ are the SDG concentrations in the IL-rich phase and salt-rich phase, respectively. The extraction efficiency (E) of SDG in the IL-rich phase was determined from Equation (4):

(4) $$\text{E}=\frac{\text{K}}{\left(\text{K}+\frac{1}{\text{R}}\right)}\times 100\text{\%}$$

### 3.4. HPLC Conditions

SDG was quantified using Dionex UltiMate 3000 LC Modules (Sunnyvale, CA, USA), and equipped with an LPG-3400 pump (Sunnyvale, CA, USA) and a UV-Vis detector (model: VWD-3400, Sunnyvale, CA, USA). A personal computer equipped with Chameleon software was used to collect and process the chromatographic data. The analyses were performed with a Promosil C18 column (250 × 4.6 mm I.D.; 5 μm, Bonna-Agela Technologies, Tianjin, China). The mobile phase of SDG analysis was composed of methanol and H_2_O (33:67; *v*/*v*) with isocratic elution. The flowrate was 1.0 mL/min, and the effluent was monitored at a wavelength of 290 nm. The column temperature was maintained 30 °C. The samples were analyzed by injecting 20 μL of the diluted sample into the HPLC. The mobile phase and samples before injection were filtered through a 0.45 μm membrane. The linear regression equation is *Y* = 63.504*X* + 0.5651 (R^2^ = 0.9995), where *Y* is the peak area and *X* is the SDG concentration. Standard solutions of SDG were diluted at concentrations ranging from 0.25 to 4 mg/mL.

## 4. Conclusions

An IL-UAE coupled with IL-ATPS methodology was developed for the extraction and purification of SDG from flaxseed. The IL [C_4_mim]N(CN)_2_ was screened as the extractant due to its exceptional extraction ability. The optimized conditions determined using RSM for IL-UAE were IL concentration 55.49% (*w*/*w*), liquid–solid ratio 24.50:1, and ultrasonic irradiation time 40 min. The maximum extraction efficiency (93.35%) of SDG in the IL-rich phase resulted when the ATPS was composed of 45.86% (*w*/*w*) [C_4_mim]N(CN)_2_ and 8.27% (*w*/*w*) Na_2_SO_4_ at 22 °C and pH 11.0. This combination of IL-UAE and IL-ATPS provides a simple, efficient, and environmentally friendly methodology for the extraction and purification of SDG from flaxseeds. This process is also expected to be useful for the extraction of other bioactive compounds.
